# Lemon juice has protective activity in a rat urolithiasis model

**DOI:** 10.1186/1471-2490-7-18

**Published:** 2007-10-05

**Authors:** Mohammed Touhami, Amine Laroubi, Khadija Elhabazi, Farouk Loubna, Ibtissam Zrara, Younes Eljahiri, Abdelkhalek Oussama, Félix Grases, Abderrahman Chait

**Affiliations:** 1 Laboratory of Animal Physiology. Ecophysiology Unit. Department of Biology, Faculty of Sciences Semlalia, University Cadi Ayyad, PO Box 2390, Marrakech, 40000, Morocco; 2 Laboratory of Anatomopathology, Avicenne Military Hospital, Marrakech, 40000, Morocco; 3 Laboratory of Biochemistry, Avicenne Military Hospital, Marrakech, 40000, Morocco; 4 Laboratory of Applied and Environmental Chemical Spectroscopy, Urolithiasis Unit, Faculty of Science and Techniques, University Cadi Ayyad, PO Box 523, Beni-Mellal, 23000, Morocco; 5 Laboratorio de Investigación en Litiasis Renal. Edificio Mateu Orfila Universitat de les Illes Balears, Ctra. de Valldemossa km 7.5, E-07071, Palma de Mallorca, Spain

## Abstract

**Background:**

The use of herbal medicines (medicinal plants or phytotherapy) has recently gained popularity in Europe and the United States. Nevertheless the exact mechanism of the preventive effects of these products is still far to be clearly established, being its knowledge necessary to successfully apply these therapies to avoid stone formation.

**Methods:**

The effect of oral lemon juice administration on calcium oxalate urolithiasis was studied in male Wistar rats. Rats were rendered nephrolithic by providing drinking water containing 0.75% ethylene glycol [v/v] (EG) and 2% ammonium chloride [w/v] (AC) for 10 days. In addition to EG/AC treatment, three groups of rats were also gavage-administered solutions containing 100%, 75% or 50% lemon juice [v/v] (6 μl solution/g body weight). Positive control rats were treated with EG/AC but not lemon juice. Negative control rats were provided with normal drinking water, and were administered normal water by gavage. Each group contained 6 rats. After 10 days, serum samples were collected for analysis, the left kidney was removed and assessed for calcium levels using flame spectroscopy, and the right kidney was sectioned for histopathological analysis using light microscopy.

**Results:**

Analysis showed that the rats treated with EG/AC alone had higher amounts of calcium in the kidneys compared to negative control rats. This EG/AC-induced increase in kidney calcium levels was inhibited by the administration of lemon juice. Histology showed that rats treated with EG/AC alone had large deposits of calcium oxalate crystals in all parts of the kidney, and that such deposits were not present in rats also treated with either 100% or 75% lemon juice.

**Conclusion:**

These data suggest that lemon juice has a protective activity against urolithiasis.

## Background

Kidney stone formation or urolithiasis is a complex process that is a consequence of an imbalance between promoters and inhibitors in the kidneys [1]. The recurrence of urolithiasis represents a serious problem as patients who have formed one stone are more likely to form another. Not all standard pharmaceutical drugs used to prevent urolithiasis are effective in all patients, and many have adverse effects that compromise their long-term use [2].

Renal calculi can be broadly classified in two large groups: tissue attached and unattached [3]. Attached calculi are mainly integrated by calcium oxalate monohydrate (COM) renal calculi, with a detectable attachment site to the renal papilla and basically consisting of a core located near to the attachment site (concave zone) and radially striated concentrically laminated peripheral layers. Unattached calculi, with no detectable site of attachment to papilla, are developed in renal cavities of low or reduced urodynamic efficacy and can exhibit diverse composition and structures. Several reports have been published since Randall's first description of papillary calcifications and their possible active role in the genesis of COM papillary calculi [4-6]. At present, it seems clear that renal epithelial cell injuries play a decisive role in such a type of renal calculi development [7,8], and in fact the lithogenic effect caused by ethylene glycol (EG) must be mainly attributed to the oxidative damage caused by the high level of oxalate generated by EC. Thus, although EC rat model can be questioned as a general model to study renal stone formation, it must be considered as an interesting model to evaluate renal papillary stone development, at least for those stones which genesis is linked to oxidative cell damage. Thus, the first studies on experimental EC renal lithiasis appeared in the 60' decade [9,10] but the importance of the oxidative damage caused by hyperoxaluria was not clearly proposed until the end of the century [11]. From this last period it appeared several prophylaxis proposals on EC induced nephrolithiasis using herbal extracts and antioxidants [12-19]. In all these papers the effects of these compounds did not seem to be mediated by diuretic or other urinary biochemical changes and positive effects on calcium oxalate lithiasis are most likely due to antioxidative effects.

To further investigate the potential of lemon juice as a therapy for lithiasis, the present study examined the effect of lemon juice on experimentally EG-induced calcium oxalate (CaOx) nephrolithiasis in rats.

## Methods

### Animals

Thirty male Wistar rats weighing approximately 280 g were acclimated for 3 days in cages before experiments commenced. Experiments were conducted in accordance with internationally accepted standard guidelines for the use of animals. Rats had *ad libitum *access to standard chow and tap water, and were kept under a controlled 12 h light/dark cycle at 22 ± 2°C.

### Ethylene glycol-induced urolithiasis

The thirty rats were divided into five groups comprising six animals per group. Each group underwent a different treatment protocol for 10 days. Group 1: negative control, *ad libitum *access to regular food and drinking water, and administered 6 μl distilled water per 1 g of body weight by gavage (intra-gastric administration). Groups 2, 3, 4 and 5: *ad libitum *access to regular food, and *ad libitum *access to drinking water containing 0.75% [v/v] ethylene glycol (EG) and 2% [w/v] ammonium chloride (AC) in order to promote hyperoxaluria and CaOx deposition in the kidneys. Groups 2, 3 and 4 were also administered 6 μl lemon juice solution/g body weight by gavage at the following concentrations: Group 2, 100% lemon juice; Group 3, 75% [v/v] and Group 4, 50%. Group 5 rats were administered 6 μl distilled water/g body weight by gavage (positive control). All rats were weighed daily.

### Assessment of antiurolithic activity

#### Kidney and serum analysis

After the 10-day experimental period, rats were anaesthetized and blood was collected from the retro-orbital region, centrifuged at 10,000 × g for 10 min [20], and the serum collected and analyzed for calcium, phosphorus, urea and creatinine using an automated system (Cobas Integra 400 plus). The rats were then sacrificed by cervical dislocation, the abdomen opened and both kidneys removed. The left kidney was dried in an oven at 100°C for 24 h, after which the kidney was weighed and then minced in a beaker containing 7 ml 0.5 N nitric acid. The mixture was then heated until the liquid became transparent. After calibration using a standard calcium solution, the calcium content of the mixture was determined using flame spectroscopy. The amount of calcium is expressed as μg/g dry kidney. The right kidney was fixed in bouin liquid [21,22], soaked in paraffin, cut at 3–4 μm intervals, and the slices stained using hematoxylin and eosin [21]. Tissue slices were photographed using optical microscopy under polarized light (Olympus BX41).

#### Statistical analysis

Results are presented as mean ± standard error (S.E.). A one-way ANOVA was used to determine the significance of differences among groups. Student's *t*-test was used to assess differences between means. Conventional Windows software was used for statistical computations. A *P *value < 0.05 was considered to indicate a significant difference.

## Results

### Serum analysis

**Figure 1:**
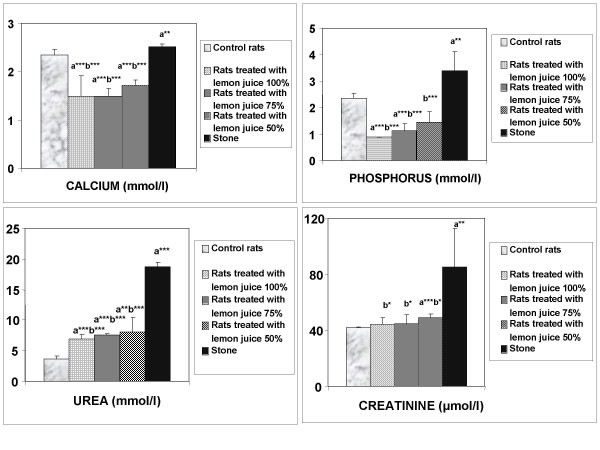
Serum biochemical data. Values represent mean ± SD for six animals in each group. ^a ^Values are significantly different from the negative control group: *p < 0.05, **p < 0.01, ***p < 0.001. ^b ^Values are significantly different from the positive control group: * p < 0.05, **p < 0.01, ***p < 0.001.

Serum analysis showed that urea and creatinine levels were higher in Groups 2, 3, 4 and 5 compared to Group 1 (Fig. 1). These data indicate marked renal damage in the EG/AC-treated rats. The data also showed that urea, creatinine, calcium and phosphorus levels were lower in rats treated with lemon juice (Groups 2, 3 and 4) compared to rats treated with EG/AC alone (Group 5, positive control).

### Body weight

**Figure 2:**
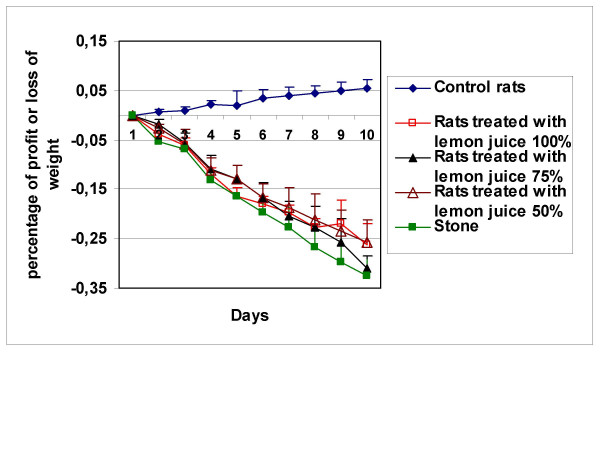
Changes in body weight in the various rat groups over the ten days of the experiment.

EG/AC-treated rats (Groups 2, 3, 4 and 5) weighed less than the negative control rats (Group 1) at the completion of the experiment (Fig. 2).

### Calcium levels in the kidneys

**Figure 3:**
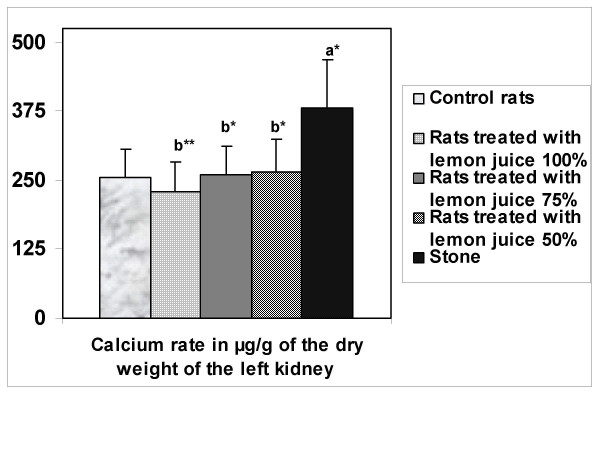
Amount of calcium in the left kidney. Values represent mean ± SD (μg/g) for six animals in each group. ^a ^Values are significantly different from the negative control group: *p < 0.05, **p < 0.01, ***p < 0.001. ^b ^Values are significantly different from the positive control group: * p < 0.05, **p < 0.01, ***p < 0.001.

The left kidneys were assessed for calcium levels. EG/AC treatment alone (Group 5) resulted in increased kidney calcium levels compared to the negative control rats, while the administration of 100% lemon juice reduced this calcium accumulation (Group 2) (Fig. 3).

### Histological examination

**Figure 4:**
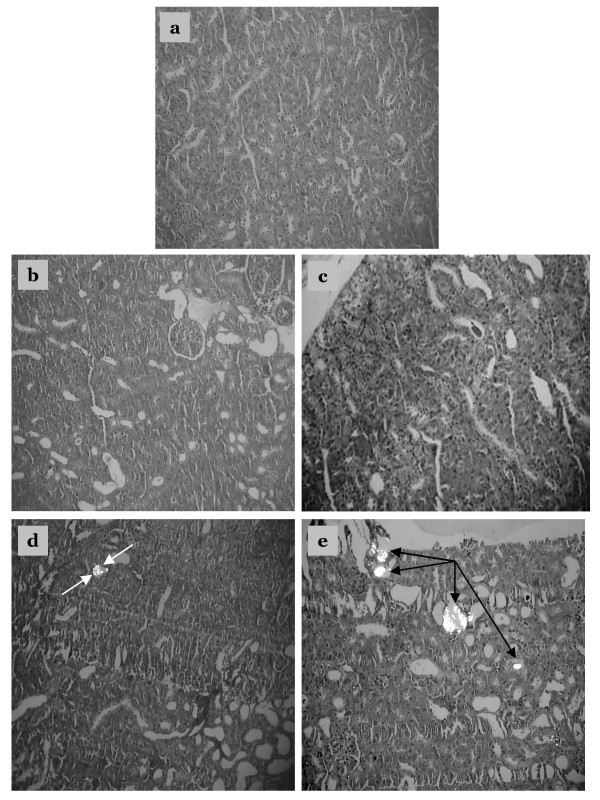
Crystalline formations in the rat kidney cortex. Sections were viewed using a BX41 optical microscope and polarized light. a: Tissue from negative control rats, b: Tissue from rats treated with ethylene glycol (EG), ammonium chloride (AC) and 100% lemon juice, c: Tissue from rats treated with EG, AC and 75% lemon juice, d: Tissue from rats treated with EG, AC and 50% lemon juice, e: Tissue from rats treated with EG and AC only (positive control). Crystalline formations in the renal cortex are indicated by arrows. Magnification ×100.

**Figure 5:**
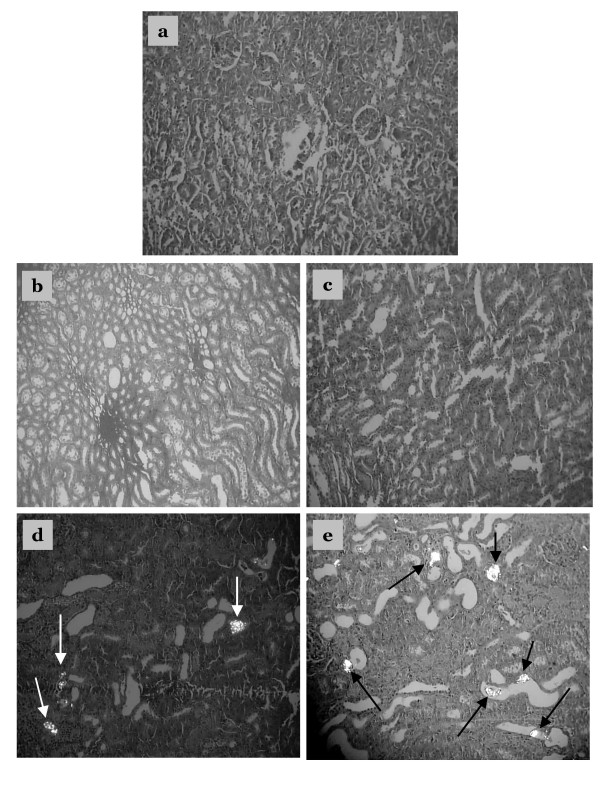
Crystalline formation in the renal parenchyma. Sections were viewed using a BX41 optical microscope and polarized light. a: Tissue from negative control rats, b: Tissue from rats treated with ethylene glycol (EG), ammonium chloride (AC) and 100% lemon juice, c: Tissue from rats treated with EG, AC and 75% lemon juice, d: Tissue from rats treated with EG, AC and 50% lemon juice, e: Tissue from rats treated with EG and AC only (positive control). Crystalline formations in the renal parenchyma are indicated by arrows. Magnification ×100.

**Figure 6:**
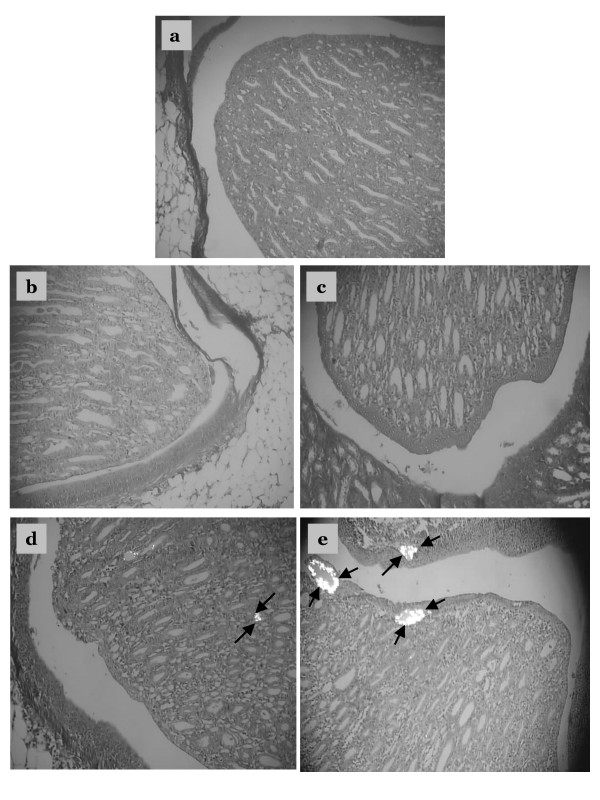
Crystalline formations in the renal papilla. Sections were viewed using a BX41 optical microscope and polarized light. a: Tissue from negative control rats, b: Tissue from rats treated with ethylene glycol (EG), ammonium chloride (AC) and 100% lemon juice, c: Tissue from rats treated with EG, AC and 75% lemon juice, d: Tissue from rats treated with EG, AC and 50% lemon juice, e: Tissue from rats treated with EG and AC only (positive control). Crystalline formations in the renal papilla are indicated by arrows. Magnification ×100.

Examination of kidney paraffin sections showed that Group 5 rats (EG/AC alone, positive control) had the greatest amount of CaOx deposition, and this was present in all parts of all three major areas of the kidney. Intratubular and interstitial crystals were observed on the cortex (Figs. 4d and 4e). There was greater calcification on surface of the renal parenchyma (Fig. 5) and the papillary tip (Fig. 6) in Group 5 rats compared to the Groups 2, 3 and 4 rats (EG/AC and lemon juice). Longitudinal sections showed the papillary tips were encrusted with CaOx crystals (Figs. 6d and 6e). Analysis of portions of these crystalline deposits removed from the papillary tip showed they were composed of CaOx monohydrate and CaOx dihydrate. No papillary encrustations were seen in tissue from the negative control rats (Group 1) (Fig. 6a) or rats treated with EG/AC and 100% lemon juice (Group 2) (Fig. 6b). Major calcium deposits were observed on the surface of the papillary tips in 33% of the positive control rats (Group 5) and 17% of the rats treated with EG/AC and 75% lemon juice (Group 3). All positive control rats (Group 5) had major calcium deposits on the surface of the cortex and medulla, while no such deposits were observed in the negative control rats (Group 1) (Tables 1 and 2). These morphological findings were consistent with the left kidney calcium level data.

**Table 1:** Number and type of calcifications observed

**Groups**	**Percentage of rats with major calcifications on the papillary tip (> 90% of the papillary tip calcified)**	**Percentage of rats with some area of the papillary tip calcified**	**Percentage of rats with some calcified points on the papillary tip**	**Percentage of rats without calcifications on papillary tip**
**1. Negative controls**	-	-	-	100
**2. EG, AC and 100% lemon juice**	-	-	-	100
**3. EG, AC and 75% lemon juice**	17	-	-	83
**4. EG, AC and 50% lemon juice**	-	-	50	50
**5. EG and AC (positive controls)**	33	33	17	17

**Table 2:** Cortex and medullar tissue data (see text for description of various groups)

**Crystal deposits**	**Group 1 n = 6**	**Group 2 n = 6**	**Group 3 n = 6**	**Group 4 n = 6**	**Group 5 n = 6**
**None**	6	5	5	-	-
**Crystals: +**	-	1	1	2	-
**Crystals ++**	-	-	-	4	-
**Crystals +++**	-	-	-	-	6

## Discussion

Urinary lithiasis is generally the result of an imbalance between inhibitors and promoters in the kidneys. Human kidney stones are usually composed of CaOx [1], and several studies have examined the effect of the citrus juices on calcium salt crystallization [23-27]. However, the conclusions from those studies were not consistent.

Many in vivo models have been developed to investigate the mechanisms involved in the formation of urinary stones, and to ascertain the effect of various therapeutic agents on the development and progression of the disease [28-33]. Rats are the most frequently used animals in models of CaOx deposition in the kidneys, a process that mimics the etiology of kidney stone formation in humans [28]. Rat models of CaOx urolithiasis induced by either EG alone or in combination with other drugs such as AC, are often used to study the pathogenesis of kidney crystal deposition [30]. Using the accelerated model [32], in the present study rats were treated with 0.75% EG and 2% AC for 10 days. All positive control rats (Group 5) developed CaOx depositions during that time.

The present study examined the effect of various lemon juice concentrations on the deposition of CaOx crystals within the rat kidney. Previous studies concluded that medicinal plants had little effect on the urinary chemistry of urolithiasis [34,35]. The current study analyzed body weight, kidney calcium level, serum concentrations of calcium, phosphorus, urea and creatinine, and the histopathology of the kidney. We found that Group 1 rats (negative controls) remained active and gained weight, while Group 2, 3, 4 and 5 rats lost weight over the 10 days of treatment. Microscopic examination using polarized light of kidney sections derived from nephrolithiasic rats showed intratubular and interstitial crystal deposits, consistent with the findings of others [36]. These crystals were intensely birefringent, polycrystalline, and arranged in a rosette characteristic of CaOx crystals. The presence of such deposits is evidence of adhesion and retention of particles within the renal tubules. These crystal deposits were observed in the kidneys of all Group 5 rats. Moreover, 33% of these rats showed major calcifications on the papillary tip. In contrast, no rats treated with lemon juice showed such papillary crystalline deposits. Rats treated with 100% or 75% lemon juice had far less kidney calcification and lower renal tissue calcium levels than the positive control rats (Group 5) (Table 1 and 2). No papillary encrustations were seen in 100%, 83% and 50% of rats treated with 100%, 75% and 50% lemon juice, respectively. Furthermore calcic parenchymatous deposits were not observed in 83% of rats treated with 100% and 75% lemon juice. These results clearly demonstrate the ability of the lemon juice to prevent the development of papillary and renal parenchymatous calcifications on the kidney, consequently preventing the development of papillary and parenchymatous calculi. All rats treated with 50% lemon juice showed fewer calcium deposits on the kidney surface than positive control rats (Group 5). While treatment with 100% and 75% lemon juice appeared to be more beneficial that treatment with 50% juice, this difference was not found to be statistically significant.

The association of crystals with renal tubular cells is considered a potential factor in the process of renal stone formation. Indeed, calculations considering the rate of crystal growth even at its maximum speed and tubular fluid flow suggest that a single crystal would not become large enough to be retained and occlude the lumen during its normal transit through the nephron [28]. Furthermore, it is established that crystals, especially calcium oxalate monohydrate crystals, can be retained by attachment to the surface of renal epithelial cells and be internalized [28].

Lemon juice has a high antioxidant capacity due to the presence of citrate, vitamin C, vitamin E and flavonoids such as eriocitrin, hesperetin [37,38] and limonoids [39]. Vitamin E may prevent calcium oxalate crystal deposition in the kidney by preventing hyperoxaluria-induced peroxidative damage to the renal tubular membrane surface (lipid peroxidation) [40,41], which in turn can prevent calcium oxalate crystal attachment and subsequent development of kidney stones [41,42].

In urolithiasis, the glomerular filtration rate (GFR) decreases due to stones in the urinary system obstructing urine outflow. This leads to the accumulation of waste products in the blood, particularly nitrogenous substances such as urea, creatinine and uric acid. In addition, increased lipid peroxidation and decreased levels of antioxidant potential have been reported in the kidneys of rats supplemented with a calculi-producing diet [20]. In this context, oxalate has been reported to induce lipid peroxidation and to cause renal tissue damage by reacting with polyunsaturated fatty acids in cell membranes [20]. In the present study, the positive control calculi-induced rats (Group 5) were found to have marked renal damage, consistent with the elevated serum levels of creatinine and urea. The administration of lemon juice inhibited these changes that would otherwise promote new stone formation in the urinary system. In rats treated with lemon juice, we attribute the lower serum creatinine and urea levels to an enhanced GFR and the anti-lipid peroxidative property of lemon juice [20]. As commended, the lithogenic effects of EG must be mainly attributed to the oxidative damage caused by the high level of oxalate generated by this substance. For this reason, the presented studies were focused to evaluate the effects on renal papillary tissue through histological studies and the protective effects caused by the consumption of lemon juice. Previous studies evaluated the effects of citrate on renal lithiasis induced by EG [43,44]. Nevertheless, to attain an increase in citrate excretion it is necessary to induce metabolic acidosis in rats and to achieve this condition it is necessary to increase the doses of EG to 2%. In such case, urinary pH of EG treated rats was clearly inferior to urinary pH of control group, the treatment with high doses of potassium citrate significantly increased the urinary pH and, as a consequence, the urinary citrate excretion notably rose. Nevertheless, EG doses of 0.75% practically did not change the urinary pH value when compared with control group [36,44] and consequently the administration of citrate did not cause important changes in urinary citrate excretion [45].

## Conclusion

The present study found that the administration of lemon juice effectively prevented the development of urolithiasis in rats. These findings support the use of lemon juice as an alternative medicine to prevent urolithiasis. Further research is necessary to clarify the mechanism underlying this preventative effect of lemon juice.

## Competing interests

The author(s) declare that they have no competing interests.

## Authors' contributions

MT participated in this study by gavage of rats, measurement of body weight and analysis of kidney calcium levels. AL performed the statistical analysis. KE participated in the animal experiments. FL participated in laboratory management. IZ examined the histological samples. YE participated in analytical determinations. AO performed image processing. FG participated in the evaluation and discussion of the obtained results. AC participated in coordination. All authors read and approved the final manuscript.

## Pre-publication history

The pre-publication history for this paper can be accessed here:


